# *Costus loangensis*, an exciting new species from Gabon, Africa

**DOI:** 10.3897/phytokeys.18.3713

**Published:** 2012-11-19

**Authors:** Hiltje Maas-van de Kamer, Paul J. M. Maas, Chelsea D. Specht

**Affiliations:** 1 Netherlands Centre for Biodiversity (section NHN) / Biosystematics Group, Wageningen University, Generaal Foulkesweg 37, 6703 BL Wageningen, The Netherlands; 2 Departments of Plant and Microbial Biology and Integrative Biology & The University and Jepson Herbaria, University of California, Berkeley, CA 94720

**Keywords:** Africa, Gabon, Costaceae, *Costus*, Zingiberales, spiral ginger

## Abstract

A new species of spiral ginger (*Costus*: Costaceae) from Gabon, Africa is described. *Costus loangensis* H. Maas & Maas is found in the coastal region on white sand soils under a tropical rain forest canopy. It is morphologically distinct from all other African species of *Costus* but shows some similarities in floral form with the savanna-inhabiting *Costus spectabilis* (Fenzl) K. Schum. and similarities in vegetative form with *Costus ligularis* Baker. Only one population of the new species is documented. Photographs of the new species are included as is a preliminary phylogeny indicating its position within the African Costaceae.

## Introduction

The plant family Costaceae is pantropical in distribution with its largest genus, *Costus* L., restricted in distribution to African and New World Tropics. Based on phylogenetic studies, the ancestral distribution of *Costus* is the African tropics and its current distribution is reflective of a long distance dispersal event from Africa to the neotropics (Specht et al. 2001). Estimates based on fossil calibrations indicate that the Neotropical lineage diverged from the African *Costus* clade approximately 33 million years ago (Specht 2006b), while subsequent diversification of the neotropical *Costus* lineage based on rates of molecular evolution is estimated to have occurred within the past 4 million years (Kay et al. 2005), indicating a recent rapid radiation of the neotropical *Costus* lineage following colonization. There are currently ~80 species in the new world clade as compared with only ~25 species in the combined African lineages.

In all *Costus* species, the staminodial labellum, formed by the fusion of five petaloid organs in the stamen whorl, is predominantly responsible for the floral display. The ancestral *Costus* flower had a broad, open labellum; solid white or yellow in color; with no strong markings indicative of a specific pollination syndrome (Specht et al. 2001). Phylogenetic studies using molecular and morphological data (Specht 2006a) indicate a single evolutionary origin of the bee-pollinated floral form from the ancestral open floral morphology, resulting in a relatively diverse African clade (~7 species) all with a floral morphology indicative of bee pollination (e.g. *Costus afer* Ker Gawl., *Costus dubius* (Afzel.) K. Schum). The Neotropical *Costus* lineage is sister to this bee-pollinated African clade, and the earliest diverging species of the neotropical clade retain the ancestral bee-pollination syndrome (Specht 2006b; Kay et al. 2005). Within the neotropical *Costus*, bird pollination has evolved as many as 7 times, each associated with a radiation of a bird-pollinated lineage (Kay et al. 2005, Specht 2006a). It is hypothesized that this evolutionary toggle between bee and bird pollination and adaptation to hummingbird pollination (Kay et al. 2005) may have led to the rapid radiation of this lineage in the Neotropics subsequent to its divergence from the African ancestral populations (Specht 2006b). There are no known bird-pollinated species in Africa, with the possible exception of *Costus giganteus* Welw. ex Ridl. from São Tomé and Principe which bears red bracts and tubular yellow flowers that resemble those of Neotropical bird-pollinated species.

As part of a larger effort to monograph all African species of *Costus*, the authors recovered a photograph of a *Costus* flower published in a guide to the Loango National Park (Vande weghe 2007). This photo represented a plant that was not present in studied herbarium or living material and had not yet been described. An expedition to Gabon with a targeted trip to the Loango National Park (= Parc Nacional de Loango) and the surrounding region revealed a single population of the unknown species, here described.

### Costus loangensis

H. Maas & Maas sp. nov.

urn:lsid:ipni.org:names:77123154-1

http://species-id.net/wiki/Costus_loangensis

#### Diagnosis.

**Figure 1.**
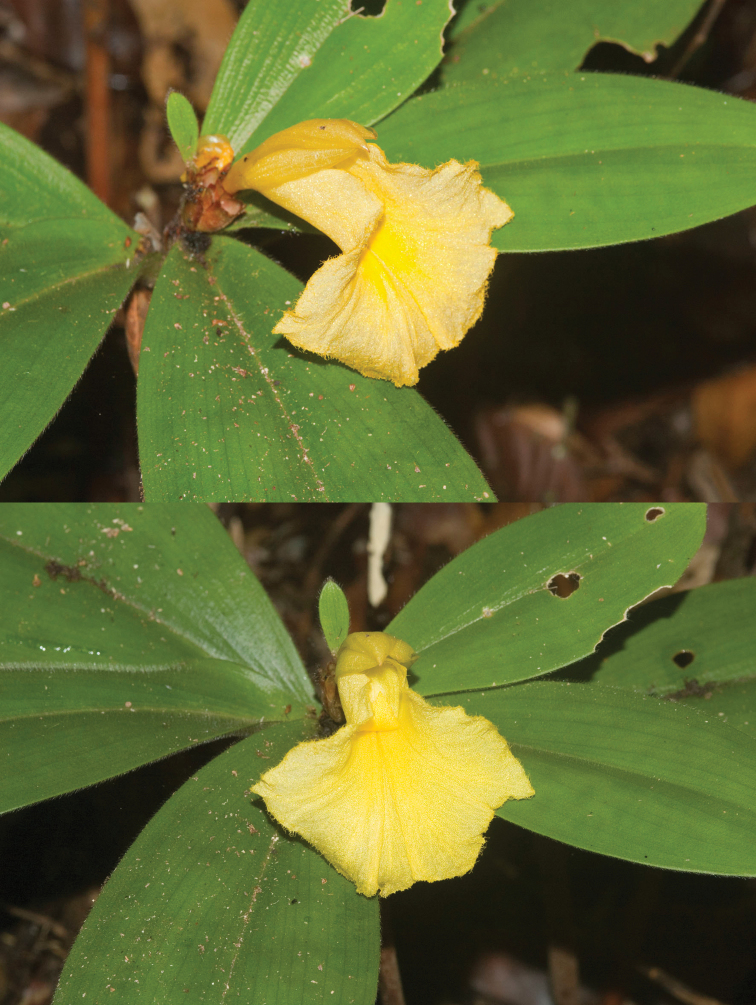
Photographs of *Costus loangensis* from the single known population. Photo credit: J.P. Vande weghe.

*Costus loangensis* (Fig. 1) is a short-stemmed (about 0.5 m tall) plant with few (6–7) leaves and with completely yellow flowers. Stems and leaves are covered with a dense indument of erect to half-appressed hairs. *Costus loangensis* differs from *Costus spectabilis* (Fenzl) K.Schum., another short-stemmed yellow-flowered species, by having a well developed aerial stem. *Costus spectabilis* inhabits savannas and has only 4 leaves that remain strongly pressed to the ground. *Costus loangensis* differs from *Costus ligularis* Baker, also a short-stemmed and indumented plant, by the colour of its flowers (*Costus ligularis* has a pale pink flower) and the length of the calyx (5-7mm in *Costus ligularis* v. 11–12mm in *Costus loangensis*) (Table 1).

**Table 1.** Use of characters to differentiate *Costus loangensis* from other African species with similar morphological characters.

**Species**	**Flower color**	**Stem height**	**Bract appendage**
*Costus loangensis*	yellow	0.5m	No
*Costus spectabilis*	yellow	0m	No
*Costus ligularis*	pale pink to white	0.5m	No
*Costus gabonensis*	yellow	1.5m	Yes, red/brown reflexed

#### Type.

*P.J.M. Maas, F.J. Breteler, C.D. Specht, H. Maas-van de Kamer, R. Niangadouma 10184* (holotype WAG; isotypes K, LBV, MO, UC), Gabon, prov. Ogooué-Maritime: Parc Nacional de Loango, between Lodge and Staff building, 1°54'43.3"S, 9°19'33.6"E, wet forest on white sand, along forest trail, at about sea level, 9 November 2011.

#### Description.

Terrestrial herb, 0.5–0.6 m tall, stems dark brownish red. *Leaves*: dark olive-green, several (6–7) concentrated at the apex of the stem; sheaths dark red, 0.6–0.8 cm diam.; ligule green, 2-lobed, 15–18 mm long, membranous; petiole 5–6 mm long; sheaths sparsely to rather densely covered with erect to half-appressed hairs ca. 2 mm long, ligule and petiole densely to rather densely so; lamina narrowly elliptic to elliptic, 14–16 by 5–6 cm, densely to rather densely covered with erect to half-appressed hairs 1.5–2 mm long on both sides, zone along midrib sometimes reddish, base attenuate, apex acute. *Inflorescence*: 3–5-flowered, ovoid, 2 by 1–1.5 cm, terminating the leafy stem; outer side of bracts, bracteoles and calyx densely covered with appressed to half-appressed hairs ca. 0.2 mm long, ovary sparsely so. *Flower*: 1 per bract; bracts brown to reddish brown, chartaceous, narrowly ovate-triangular to ovate-triangular, 1.7–2 by 0.5–1 cm, callus 2.5–3 mm long; appendages absent; bracteole reddish, boat-shaped, 15–18 mm long, callus 1.5–2 mm long; calyx reddish to greenish, 11–12 mm long, lobes deltate, ca. 2 mm long, callus ca. 1 mm long; corolla yellow, 50–55 mm long, tube 20–25 mm long, lobes narrowly elliptic, 30–35 mm long, outer side rather densely covered with half-appressed hairs ca. 1 mm long particularly near the apex, together forming a hood over the throat opposite the labellum, apex with a callus-like thickening; labellum yellow, horizontally flattened with funnel-shaped base, broadly obovate when spread out, 30–40 by 40–50 mm, margin fimbriate (fimbriae 2–3 mm long); stamen yellow, 25–30 by 7–10 mm, apex reflexed, anther 5–7 mm long; ovary narrowly obovoid, ca. 6 mm long, stigma bilamellate, dorsal appendage 2-lobed. *Fruit* and *seeds* not seen.

#### Distribution.

**Figure 2.**
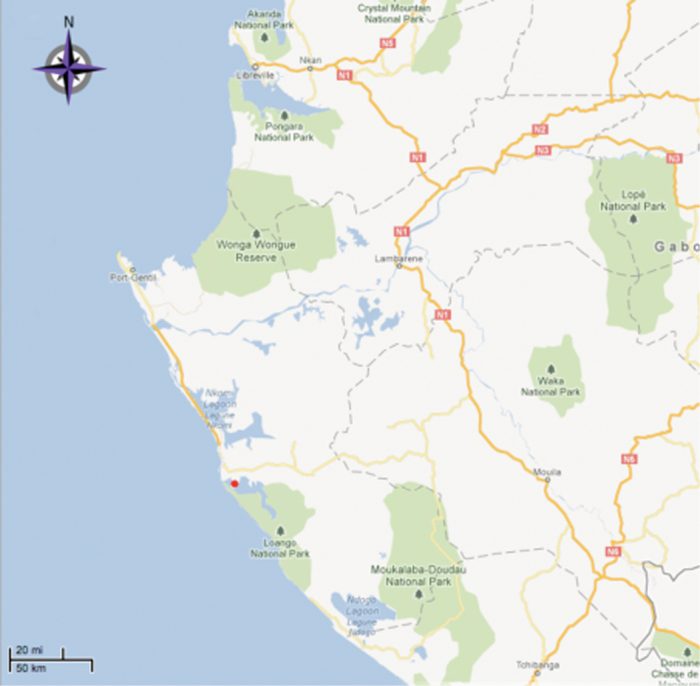
Map of locality for *Costus loangensis*. The red dot represents the type locality.

Gabon (Fig. 2). Only known from the type location.

#### Habitat and ecology.

**Figure 3.**
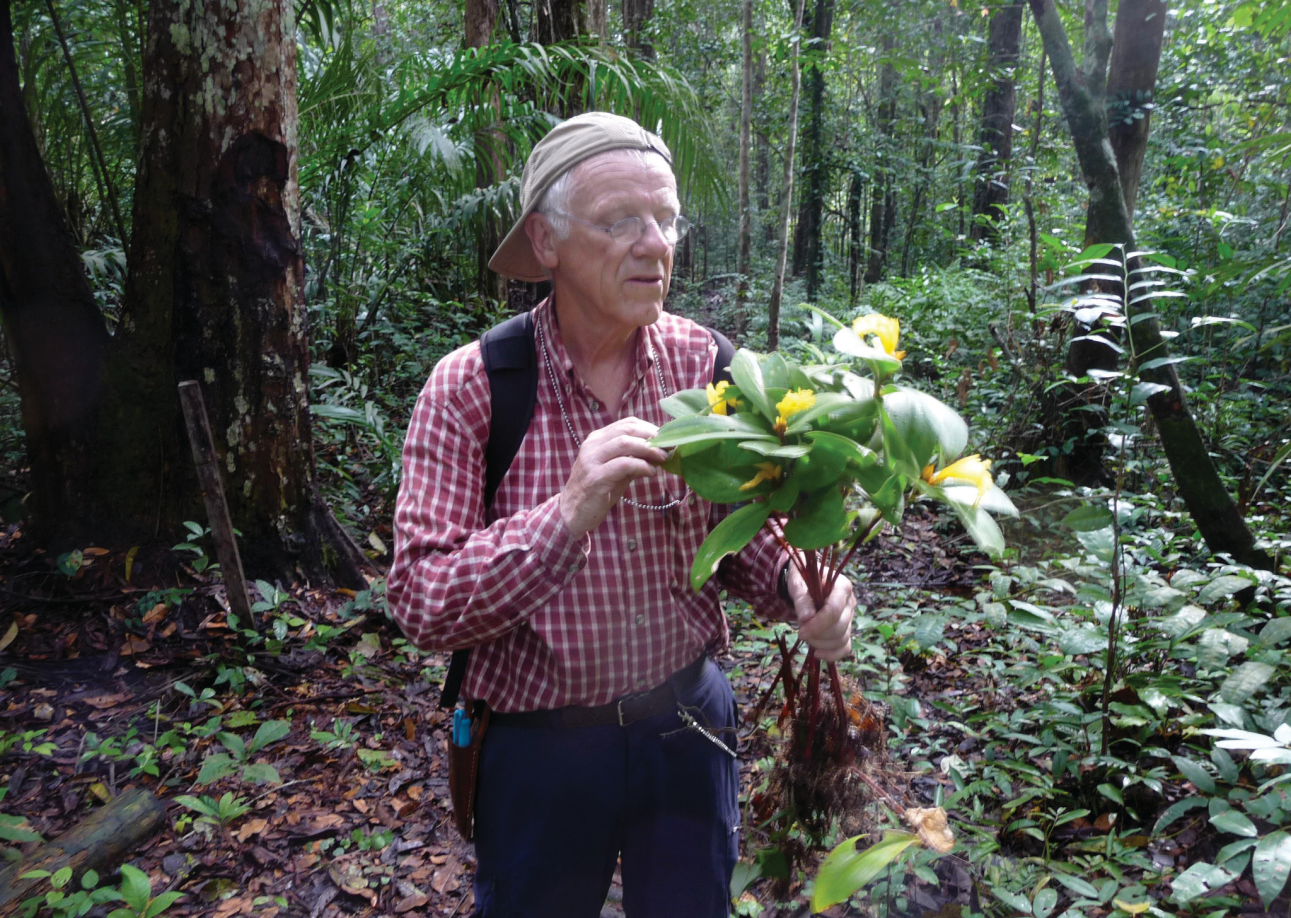
*Costus loangensis* habit and habitat, as being studied by co-author P.J.M. Maas. Photo credit: H. Maas-van de Kamer.

In wet forest, on white sand soil under rain forest canopy (Fig. 3). Elevation just above sea level. Flowering: November; fruiting: unknown.

#### Phylogenetic relationship.

**Figure 4.**
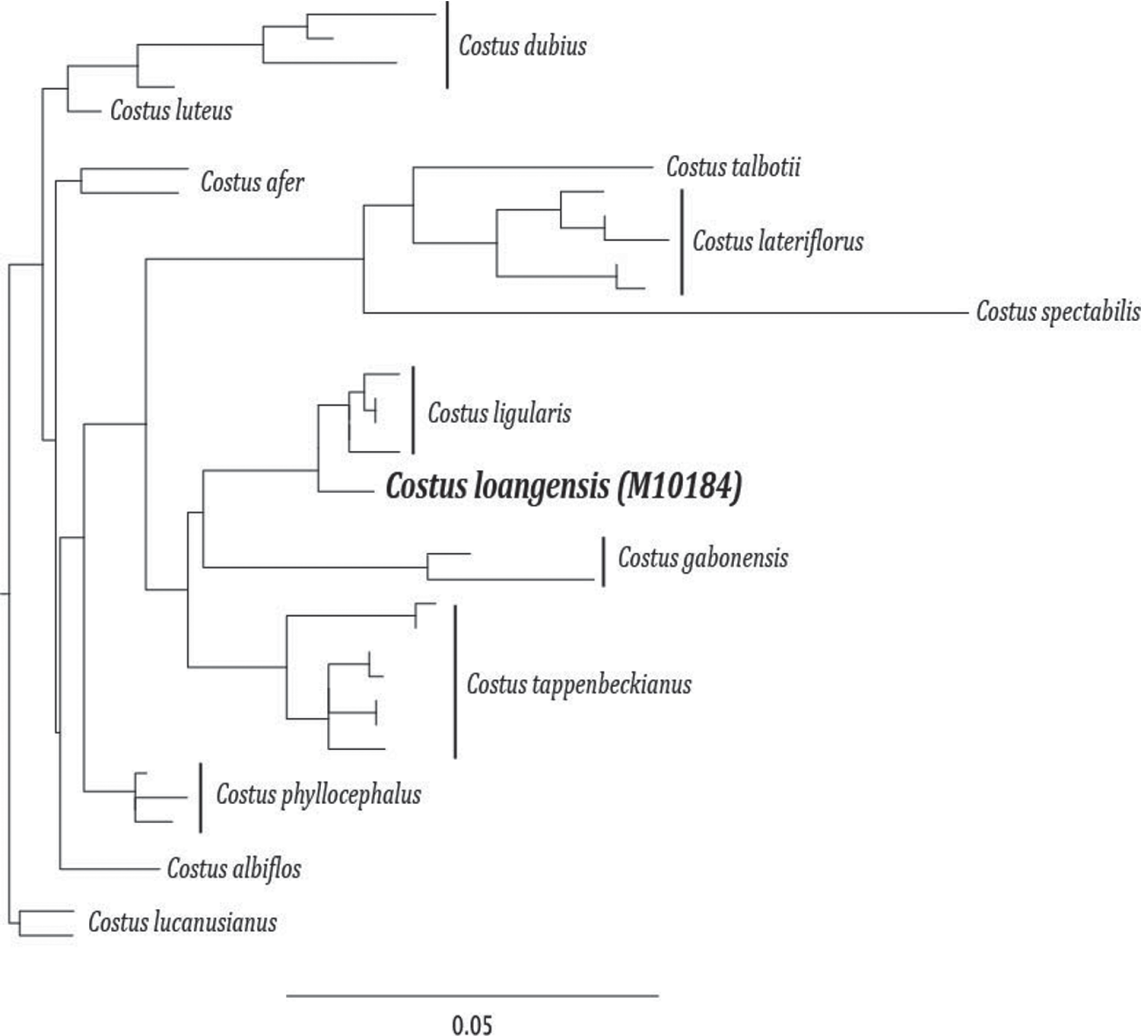
Phylogeny of African *Costus* species (Table 2) including newly described *Costus loangensis*. The phylogeny was constructed in PhyML (Guindon and Gascuel 2003) with aligned sequence data for two low copy nuclear markers [calmodulin (cam) intron (Johansen 2005) and rpb2 (Specht 2006a)] and nrDNA markers ITS and ETS. All nodes indicated have >50% bootstrap support.

Based on a combined 4 molecular marker (CAM, ITS, ETS, rpb2) phylogenetic analysis with taxon sampling that included African species in the genus *Costus*, *Costus loangensis* H. Maas & Maas is found to be well supported as sister to a clade of *Costus ligularis* Baker specimens (Fig. 4). *Costus loangensis* fits within the general distribution range of *Costus ligularis*, however no populations of *Costus ligularis* were found in sympatry. *Costus loangensis* is restricted in elevation to just above sea level, while *Costus ligularis* is commonly found 0–600m above sea level throughout low-elevation rain forests in Cameroon and Gabon.

**Table 2.** List of collections included in phylogeny to place *Costus loangensis* sp. nov.

*Costus*	*Costus afer*	L87-0185
*Costus*	*Costus afer*	M10205
*Costus*	*Costus albiflos*	M10411
*Costus*	*Costus dubius*	M10206
*Costus*	*Costus dubius*	GH89-0918
*Costus*	*Costus aureus*	M9302 (vouchered from Burger’s Bush)
*Costus*	*Costus lucanusianus*	M10000
*Costus*	*Costus lucanusianus*	L87-0286
*Costus*	*Costus ligularis*	M10329
*Costus*	*Costus ligularis*	M10267
*Costus*	*Costus ligularis*	BB 1998-0923003
*Costus*	*Costus lateriflorus*	M9995
*Costus*	*Costus lateriflorus*	M10331
*Costus*	*Costus lateriflorus*	GH98-224
*Costus*	*Costus spectabilis*	GH96-284
*Costus*	*Costus gabonensis*	M10291
*Costus*	*Costus gabonensis*	CS02-339
*Costus*	*Costus tappenbeckianus*	M10226
*Costus*	*Costus tappenbeckianus*	GH94-628
*Costus*	*Costus talbotii*	BB 2003-0109009
*Costus*	*Costus phyllocephalus*	M10389
*Costus*	*Costus phyllocephalus*	L87-0057
*Costus*	*Costus phyllocephalus*	BB 2001-0402004

L=Lyon Arboretum M=Maas collection CS=C. Specht collection GH=Greenhouse at Smithsonian NMNH BB=Burger’s Bush

#### Vernacular names.

Not recorded. This plant seems to go unnoticed by the local population and has no known uses.

#### Etymology.

*Costus loangensis* is named after the place where it was photographed and now has been collected and documented for the first time: near the Loango Lodge, in “Parc Nacional de Loango” in Gabon.

## Supplementary Material

XML Treatment for Costus loangensis
